# News and Miscellany

**Published:** 1901-07

**Authors:** 


### News and Miscellany.

---

**A young physician** with over a year's experience in hospita practice would like to assist and office with a busy practitioner in a large town or city.

H. J. Warner, M. D., Stamford, Texas.

---

**There is room** in the State Lunatic Asylum at Austin for 200 additional patients. The Governor is advertising the fact, and county judges and physicians will take notice. Indigent patients have preference.

**The M. J. Breitenbach Co.,** 53 Warren Street, New York City, representatives of Gude's Pepto-Mangan, most cordially invite all of the medical profession, when visiting New York City, to make their office a business home.

---

**Married,** at Brenham, Texas, June 18, Dr. W. A. Harper, of Austin, to Miss Alice Baker, of Brenham. Dr. Harper is a popular oculist of Austin, and is secretary of the Austin District Medical Association. We extend congratulations to him.

---

**Pierpont Morgan** gave Harvard Medical College a million for a biological laboratory. The University of Pennsylvania is building one to cost half a million and Rockefeller gave New York $200,000 for bacteriological research. The microbes are put upon notice that we are onto them.

---

**The seventh quarterly** meeting of the Panhandle Medical Association will meet in Amarillo July 16 and 17. A splendid program has been prepared and a large attendance is expected. Dr. D. R. Fly, Amarillo, president and Dr. T. W. Carroll, Clarendon, secretary.

---

**The Central Texas Medical Association**, Dr. N. A. Olive president, held its regular semi-annual meeting at Temple on 9th inst. The JOURNAL did not get out in time to make it worth while to publish the program; and got out too early to publish the proceedings, not yet to hand.

---

**The Austin District Medical Society** held its regular June meeting in Austin on 14 and 15 ult., but as the secretary went off soon afterwards and got married, or got married and went off, or both, he didn't furnish the "Red Back" with any report of proceedings or any of the papers.

---

**So Wyeth** got there. Glad indeed to know it. But the *Medical Mirror* says "his interests were under the able management of Dr. Harris." We don't like the idea of electing a president of the A. M. A. by the methods used in politics. The honor should be spontaneous. The idea of "running for president" is a little—well, I don't like it.

---

**Physician's Practice for Sale.**—Good practice in railroad town of 500 inhabitants, good paying German community, South-central Texas. No competition. Purchaser pays forp roperty only. Will introduce and recommend purchaser. If you mean business, address Dr. X., TEXAS MEDICAL JOURNAL, Austin, Texas.

**A physician,** graduate of a reputable medical college, with hospital experience and some experience in general practice, desires to associate himself with an older physician either in town or in a thickly settled country community. Single. Good moral character. Can give references. Address "Doctor," care TEXAS MEDICAL JOURNAL.

---

**New Orleans Polyclinic.**—Fifteenth annual session opens November 4, 1901. Physicians will find the Polyclinic an excellent means for posting themselves upon modern progress in all branches of medicine and surgery. The specialties are fully taught, including laboratory work. For further information address Dr. Isadore Dyer, Secretary, New Orleans Polyclinic, postoffice box 797, New Orleans, La.

---

**Students** who will attend lectures this fall will do well to carefully look over the advertisements of the many medical colleges in this issue, and write to the secretary of each one for further information. They are all our friends and patrons, and while we would like to say something nice about them we should have to say something nice about each one, we could not discriminate, and that would not be worth a cent to either one. Best way is to go to the dean for information.

---

**And old Saunders,** Dudley D. the grim old veteran, was elected president of the Association of the Army and Navy Surgeons of the Confederate States. I'll have to celebrate on the strength of the news. Keller's retiring address was grand. So was Erskine's address of welcome. Subscribe for Old Roberts' *Nonpareil*, the *Southern Practitioner*, the official organ U. C. V., and get the record with the handsome picture of Saunders, July number.

---

**Amendment of Constitution and By-Laws,** Texas State Medical Association. The following have been appointed by the president of the State Medical Association a committee on revision of the Constitution and By-Laws, to report at next meeting: Drs. J. T. Wilson, Sherman; R. F. Miller, Sherman; S. C. Red, Houston; H. A. West, Galveston; J. E. Gilcreest, Gainesville. The object is to make the Constitution and By-Laws harmonize with those of the American Medical Association.

---

**Death of Dr. Chilton.**—Dr. R. H. Chilton of Dallas, died at his home in that city June 5, after a brief illness, aged 57. Dr. Chilton was one of the most distinguished specialists (oculist) in the South. He was an old member of the State Medical Association and never missed a meeting. He was once first vice president. His friends were numbered by the thousand, and he will be greatly missed by the profession. The Dallas County Medical

Association, of which he was a member and one of the organizers twenty years ago, adopted lengthly resolutions upon his death, paying his memory a high and deserved tribute.

---

**Phenacetin Patent Sustained.**—The United States Circuit Court, Eastern District of Pennsylvania, in the case of E. N. Dickerson and Farbenfabriken of Elberfeld Company, complainants, vs. Conrad D. Maurer, defendant, has handed down a decision sustaining the suit of complainants, and issued an injunction restraining defendant from the use of the name and the manufacture of Phenacetin.

The Farbenfabriken of Elberfeld Company have issued a notice to the drug trade, from which we extract the following, that physicians too may be on their guard:

"While this case, which was understood to be a test case, involving the merits of our patent, was in progress, many infringers took advantage of the situation to offer the retail druggists of the country infringing phenacetin at low prices.

Some of these infringers have also sent circulars to the retail trade.

We regret to say that, notwithstanding our repeated warnings, some retailers have been foolish enough to purchase from these infringers, who have endeavored to mislead you in regard to our rights.

We now notify you, once for all, that having given full warning to the trade, we expect them to respect our patent, as we intend to enforce our rights under it to the full extent of the law.

While we have been compelled to institute proceedings against many druggists in the past, and have evidence that may call for many other cases, we are disposed to adjust differences with those who have infringed in times gone by, upon reasonable terms, and will be glad to correspond with any druggist with reference thereto.

Farbenfarbriken of Elberfeld Company,
40 Stone Street.

